# Supplementary figures and images for: An Intermittent Fasting Mimicking Nutrition Bar Extends Physiologic Ketosis in Time Restricted Eating: A Randomized, Controlled, Parallel-Arm Study
Source: Nutrients. 2021 Apr 30;13(5):1523. doi: 10.3390/nu13051523 (PMC8147148; doi:10.3390/nu13051523)

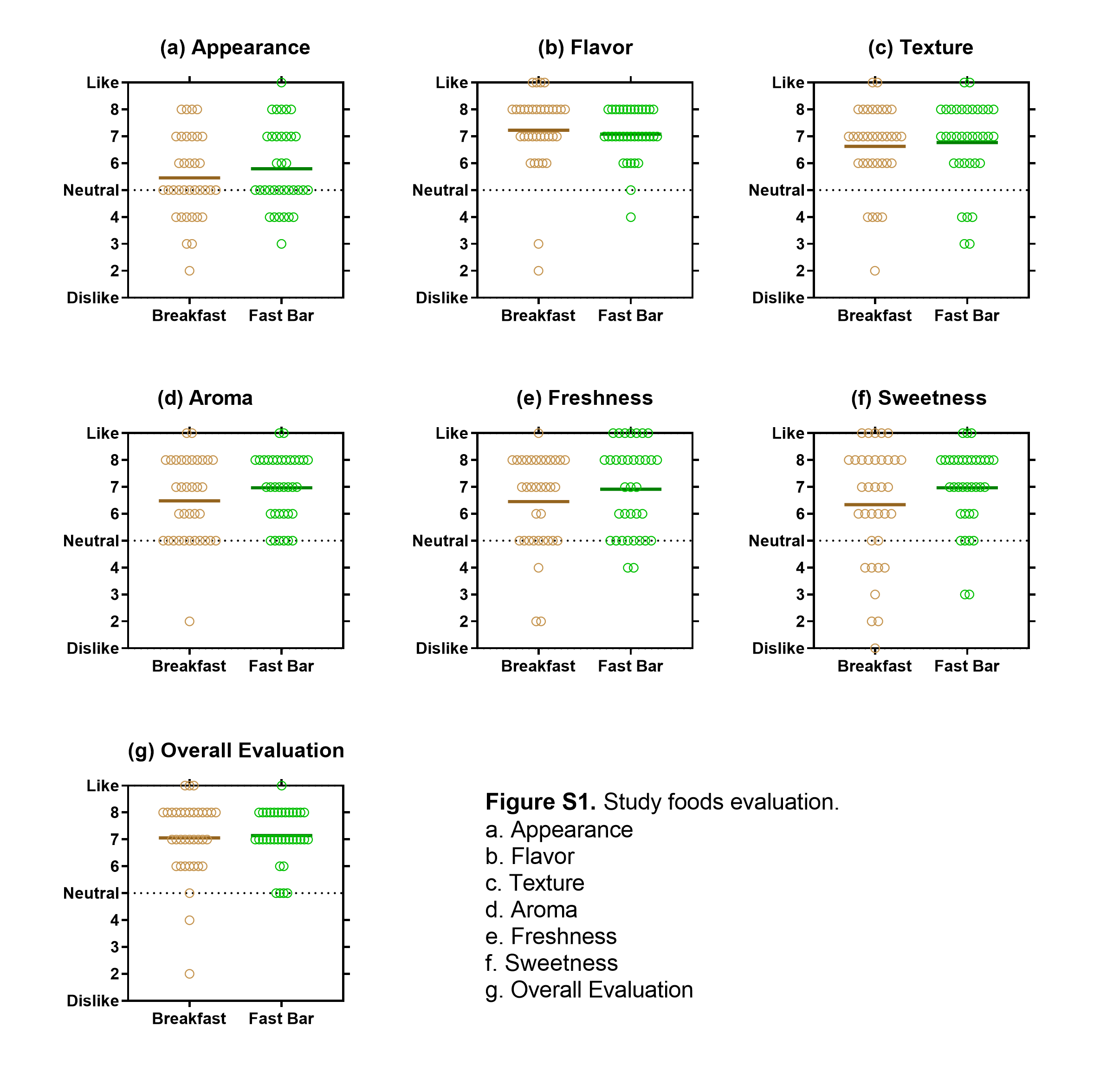

Supplement: Supplementary file 1 [file nutrients-13-01523-s001.zip › FigureS1. StudyFoodsEvaluation.tif]

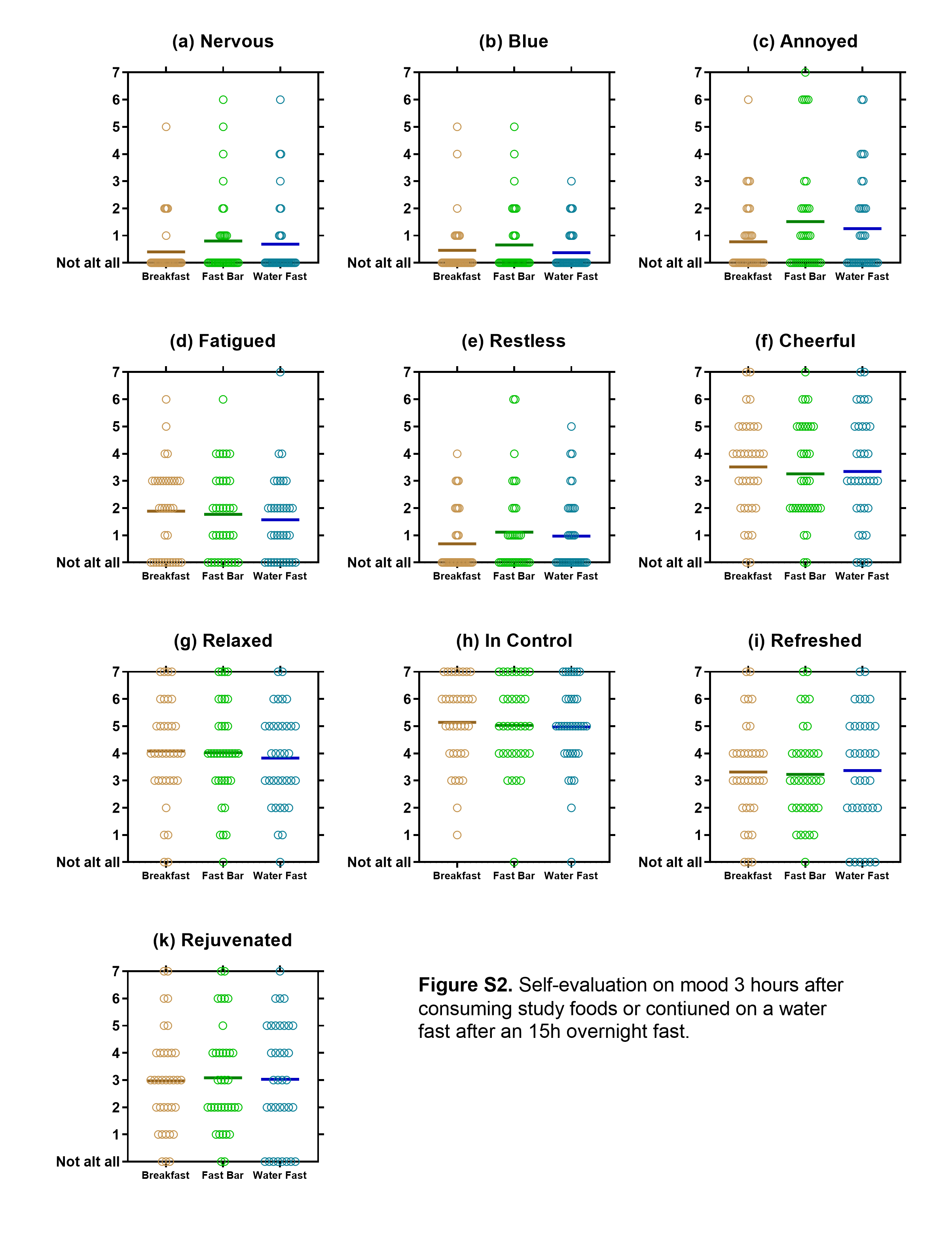

Supplement: Supplementary file 1 [file nutrients-13-01523-s001.zip › FigureS2_SelfEvaluationMood.tif]
